# Fused Filament Fabrication of Short Glass Fiber-Reinforced Polylactic Acid Composites: Infill Density Influence on Mechanical and Thermal Properties

**DOI:** 10.3390/polym14224988

**Published:** 2022-11-17

**Authors:** Lucia-Antoneta Chicos, Mihai Alin Pop, Sebastian-Marian Zaharia, Camil Lancea, George Razvan Buican, Ionut Stelian Pascariu, Valentin-Marian Stamate

**Affiliations:** 1 Department of Manufacturing Engineering, Transilvania University of Brasov, 500036 Brasov, Romania; 2 Department of Materials Science, Transilvania University of Brasov, 500036 Brasov, Romania

**Keywords:** material extrusion, fused filament fabrication, unmanned aerial vehicle, mechanical properties, infill density, thermal properties

## Abstract

Fused Filament Fabrication (FFF) is one of the frequently used material extrusion (MEX) additive manufacturing processes due to its ability to manufacture functional components with complex geometry, but their properties depend on the process parameters. This paper focuses on studying the effects of process parameters, namely infill density (25%, 50%, 75%, and 100%), on the mechanical and thermal response of the samples made of poly(lactic acid) (PLA) reinforced with short glass fibers (GF) produced using FFF process. To perform a comprehensive analysis, tensile, flexural, compression, differential scanning calorimetry (DSC), and thermal gravimetric analysis (TGA) tests were used. The paper also aims to manufacture by FFF process of composite structures of the fuselage section type, as structural elements of an unmanned aerial vehicle (UAV), and their testing to compression loads. The results showed that the tensile, flexural and compression strength of the additive manufactured (AMed) samples increased with the increase of infill density and therefore, the samples with 100% infill density provides the highest mechanical characteristics. The AMed samples with 50% and 75% infill density exhibited a higher toughness than samples with 100% infill. DSC analyses revealed that the glass transition (Tg), and melting (Tm) temperature increases slightly as the infill density increases. Thermogravimetric analyses (TGA) show that PLA-GF filament loses its thermal stability at a temperature of about 311 °C and the increase in fill density leads to a slight increase in thermal stability and the complete degradation temperature of the AMed material. The compression tests of the fuselage sections manufactured by FFF made of PLA-GF composite showed that their stiffening with stringers oriented at an angle of ±45° ensures a higher compression strength than the stiffening with longitudinal stringers.

## 1. Introduction

Material extrusion (MEX) is one of the most widely used additive manufacturing (AM) primary process classes. The standard ISO/ASTM 52900-21 [1] defines MEX as “the process in which material is selectively dispensed through a nozzle or orifice”. FFF, as a process which belongs to the MEX class, allows building of 3D objects by forcing a polymeric filament to pass through a hot nozzle and melted material is selectively deposited layer upon layer on a platform. The FFF is one of the most well-known and used process in the extrusion of thermoplastic material due to the fact that it is considered for the industrial sector a technology that allows the manufacture of components with a high geometric complexity and also, due to a wide range of thermoplastic and composite materials available that can be used in the manufacturing process. The most well-known thermoplastic materials used in FFF are polyamides (PA), acrylonitrile butadiene styrene (ABS), polycarbonate (PC), polylactide acid (PLA) etc. [2,3,4]. Polylactic acid (PLA), a thermoplastic polyester obtained from renewable sources, presents high strength, high modulus, as well as good processing capacity [5,6]. However, PLA presents some disadvantages such as brittleness and poor thermal resistance which significantly limits its use in many fields. Thus, improving the mechanical performance and thermal resistance of PLA has always been a concern in both academic and industrial areas. It was found that the poor crystallization ability of PLA is the main cause of its poor mechanical performance [5,6] and therefore concerns have been focused on improving the crystallization of PLA by reinforcing it with nucleating agents. It has been shown in [2,5,6] that fibers and nucleating agents can not only effectively promote crystallization, but also significantly improve matrix properties. In general, the fibers used to reinforce PLA can be natural fibers and synthetic fibers as also mentioned in [6,7]. Synthetic fibers include glass fiber (GF), aramid fibers, liquid crystalline polymer, and carbon fiber (CF) and natural fibers can be wood, bamboo, flax, jute, cellulose, hemp etc. [4,7,8]. It has been demonstrated in published studies [5,9,10] that PLA composite reinforced with synthetic fibers has superior characteristics (lightweight, corrosion resistance, chemical resistance, superior stiffness and strength) than those with natural fibers. Among the synthetic fibers, glass fiber is the most used as a reinforcing agent due to its superior mechanical properties, good heat resistance and also low costs as shown in previous studies [4,11,12]. An important feature of FFF technology is its ability to be used in the manufacture of light structures by modifying/optimizing the infill density of the structures. Infill density has a significant role in reducing weight, which together with materials with high strength and stiffness allow obtaining some functional structures [3,5]. Regarding glass fiber reinforced composite, most research is focused on polymers such as polypropylene (PP), polyethylene terephthalate (PET) and polyamide (PA), there being very little research on glass fiber reinforced PLA composite, in general, and extremely few regarding the influence of infill density on the mechanical and thermal characteristics of glass fiber reinforced PLA composite.

In the paper [5] it is indicated that thermal treatment can improve the tensile strength, flexural, and impact strength of PLA reinforced with glass fibers (GF) and GF can enhance crystallization of PLA. In their paper, Jing et al. [10] investigated the reinforcement of PLA with three types of glass fiber (GF): neat GF, silane-modified GF, and graphene oxide-coated GF. Glass fiber modification was found to influence matrix crystallinity, interfacial interaction which in turn affects the mechanical performance of PLA/GF composites. In [13,14] the effects of PLA reinforcement with glass fiber and natural fiber were analyzed and it was found that glass fiber reinforced PLA composite has better strength, rigidity and toughness than PLA reinforced with cellulose fiber. Varsavas et al. showed in their work [15] that glass fiber determined the increase in strength and elastic modulus values of PLA and the optimal content of glass fiber in the matrix is 15 wt%. It is well known that mechanical properties are essential for functional structures/parts and therefore it is absolutely necessary to analyze the influence of the parameters used in the FFF process on them [16]. Gomez-Gras et al. studied in [17] the influence of four factors (layer height, fill density, nozzle diameter and velocity) on the fatigue properties of the samples made of PLA and found that infill density is the most influential parameter on fatigue life, followed by layer height. In [18], the authors studied the influence of infill density on microstructure and flexural behavior of AMed PLA samples and found that bending properties are strongly influenced by infill percentage. Pandžić et al. concluded in [19] that infill density and infill type influence on ultimate tensile strength and yield strength of PLA material namely the ultimate tensile and yield strength increase as the infill density increases. In [20], Ma et al. investigated the effect of infill density and pattern of AMed cubes, made of PLA and PLA reinforced with carbon fiber, under quasi-static axial compressive loading. They found that higher infill density of AMed PLA cubic structure provides better energy-absorbing properties and the infill pattern, infill density, and material type have an important effect on crashworthiness characteristics. In the work [21], authors analyzed the influence of infill pattern and density for PLA produced by additive manufacturing (AM) technology and concluded that the flexural and compressive strength of PLA AMed material increases linearly as infill density increases. Gonabadi et al. [22] investigated the effect of build orientation, infill density and infill pattern on the mechanical properties of PLA samples obtained by AM and found that the tensile strength and Young’s modulus increase in a quadratic manner as infill density increases. Results presented in the work [23] indicated that infill density and infill pattern significantly influence the tensile strength of PLA samples produced by AM and authors found that the tensile strength increases with increment of infill density. Akhoundi and Behravesh investigated in [24] the effect of infill patterns and infill density on the tensile and flexural strengths and concluded that the higher infill density gave higher values for tensile and flexural strength, and modulus. Wang et al. [25] found that the tensile strength, elastic modulus, and elongation at break increase as the infill percentage increases. In [26], authors investigated the effects of process parameters (infill density, infill speed and pattern, nozzle temperature) and material type on mechanical, thermal and physical characteristics of AM samples and showed that Young’s modulus increased with the increase of infill density and DSC results confirmed that the mechanical properties are not directly influence by crystallinity of the PLA samples fabricated by AM. Cwikła et al. showed in [27] that the solid layers, infill density and infill pattern influence the mechanical properties of samples produced by AM using the FFF. The authors found that increased infill percentage reduced deformation and the shell thickness had a large influence on the tensile strength. Compression tests of PLA samples fabricated by AM were performed by Singh Mehta et al. [28]. The authors showed that the compressive strength of PLA samples increases with increasing filling density, shell thickness and layer height. A numerical and experimental study on the compression properties of PLA produced by FFF process is presented in [29] and Abbas et al. [30] investigated the effect of infill density on the compression properties of PLA samples fabricated by the same technology. The results showed that the high mechanical strength is achieved by using a high infill density. In [31], the authors analyzed the effect of infill line distance of FFF-fabricated circular PLA samples on their elastic compressive behavior. It was found that through a combined elastic and plastic deformation the mechanical properties such as stiffness can be changed. In [32], the thermal, rheological and crystallization properties of PLA are analyzed, as well as the technologies for obtaining it. Carrasco et al. [33] investigated the thermal stability, crystallinity and mechanical properties of PLA after processing by thermoplastic methods. The authors found that after mechanical processing, the chemical composition of PLA did not change and the crystalline structure disappeared and it was recovered after annealing. The compressive property of the PLA sandwich structure fabricated by AM was investigated in [34], and the authors found that the infill density, layer thickness, AM orientation have the greatest influence on the compressive strength. Torre et al. [35] investigated PLA produced by FFF process under compression with a special focus on buckling. The authors showed that the compressive mechanical properties in the out-of-plane AM direction differ from the tensile properties in the same direction. Zhu et al. [36] evaluated the effect of AM speed, layer height and infill density on tensile and compressive strength. Both mechanical strengths were found to have the highest values with thin layers, low print speeds and 100% infill. Baich et al. [37] tested specimens with different infill densities at tensile and compression and found that as the infill density increases the tensile stress and strain increase but the compressive stress decreases. In [38], tensile and compression tests performed on PLA material, fabricated by AM, confirmed the different behavior of AMed PLA material in tensile and compressive states.

As it was shown in previous studies, the FFF parameters including infill density can influence tensile, flexural and compressive properties but many of these studies have only been done with neat PLA. There is little information on the evaluation of the mechanical and thermal properties of glass fiber reinforced PLA and extremely few published results of the use of glass fiber reinforced PLA in composite structures and produced by FFF process. It is well known that for structural applications composite structures could be subjected to different loadings. In addition, most of the previous research of glass fiber reinforced PLA focuses on the study of FFF parameters on the mechanical properties of AM parts, with less attention on the effect of infill density. This paper focuses on the effect of infill density on the mechanical and thermal properties of PLA samples reinforced with short glass fiber. The paper also aims fabrication by FFF and compression mechanical testing of the composite structures of fuselage sections type used in the manufacturing of an unmanned aerial vehicle (UAV). It should be emphasized that the filament made of PLA reinforced with glass fiber (Filaticum Glass Reinforced) is extremely little studied and its manufacturer does not provide sufficient data on its behavior both from a mechanical and thermal point of view.

## 2. Materials and Methods

### 2.1. Design of Samples and Fuselage Sections

**Figure 1:**
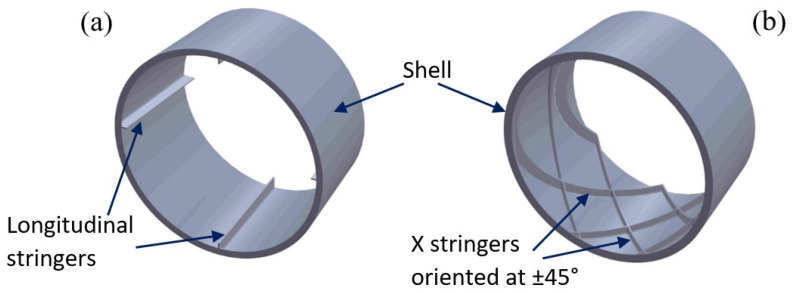
Composite structures of the fuselage section type. (**a**) fuselage section with straight (longitudinal) stringers; (**b**) fuselage section with stringers in two directions at ±45°.

**Figure 2:**
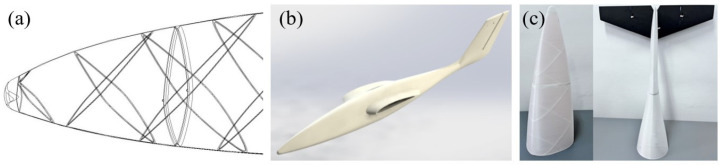
3D model of UAV fuselage. (**a**) 3D transparent view; (**b**) rendered 3D fuselage; (**c**) AMed fuselage sections.

The samples as well as the fuselage sections subjected to mechanical tests were designed in SolidWorks 2021 (Dassault 146 Systèmes SolidWorks Corporation, Waltham, MA, USA) according to ASTM standards. The Type I, dog-bone shaped, samples were used for tensile test, according to ASTM D638 [39,40], and for three-point flexural test, samples were designed using ASTM D790 [41]. ASTM D695 [42] was applied for compression tests of the rectangular samples with the size of 12.7 × 12.7 × 25.4 mm with the square cross-section. The composite structures of fuselage section type were designed and manufactured with structures (stringers) positioned in two ways: along the fuselage section (longitudinal stringers), without an angle of inclination (Figure 1a) and stringers oriented in two directions at an angle of ±45° (X stringers), for manufacturing by additive technologies (FFF) without material support (Figure 1b). Also, stringers have the role of stiffening the sections and implicitly the entire fuselage of the UAV (Figure 2). The sections of the fuselage are provided with frames with a height of 3 mm and a thickness of 1 mm (for an easier bonding between them), frames that also have the role of stiffening. The fuselage sections have the shape of the conical frustum and their dimensions and those of the stringers are shown in Table 1.

### 2.2. FFF of Samples and Fuselage Sections

The material used for AM by FFF process, both of the samples and the fuselage sections, is a filament of PLA reinforced with 20% glass fiber (GF) (Filaticum Glass Reinforced, manufactured by Filaticum, Miskolc, Hungary) [43,44]. The technical characteristics for raw and AMed material are presented in the datasheet provided by manufacturer [44]. The 3D samples and fuselage sections were fabricated on a CreatBot DX-3D double-nozzle printer (manufacturer Henan Suwei Electronic Technology Co., Ltd., Zhengzhou, China) with the parameters shown in Table 2. For each infill percentage (25%, 50%, 75%, 100%), 5 samples were fabricated by FFF process using a rectangular infill pattern. 5 samples of each infill percentage were subjected to tensile, flexural, and compression tests. Also, 5 fuselage sections were subjected to compression. Regarding the DCS and TGA analyses, one sample of each infill percentage was analyzed.

### 2.3. Testing of Samples and Fuselage Sections

Samples were subjected to tensile tests (at a crosshead speed of 5 mm/min and according to the ASTM D638 [39]), flexural (at a crosshead speed of 10 mm/min and according to the ASTM D790 [41]) and also compression (with a crosshead speed of 10 mm/min according to the ASTM D695 [42]) using W-150 S universal testing machine. Fuselage sections were fabricated using an infill density of 100% and its compression properties were also tested by using a W-150 S universal testing machine at a crosshead speed of 10 mm/min. To highlight glass transition, melting and crystallization of the as-received filament and extruded material, Differential Scanning Calorimetry (DSC) was carried out under the nitrogen atmosphere using NETZSCH DSC 200 F3 Maia (NETZSCH-Gerätebau GmbH, Germany). DSC analysis were performed in accordance with ASTM D3418 [45] at a rate of 10 °C/min, cooling to −150 °C and then heating to 500 °C. For analyzing the degradation temperature and weight loss, Thermogravimetric Analysis (TGA) were performed on the NETZSCH TG STA 449F3 Jupiter (NETZSCH-Gerätebau GmbH, Selb, Germany). TGA was conducted from 20 °C to 500 °C at a heating rate of 10 °C/min according to the ASTM Standard E1131 [46]. For the DSC and TGA analyses the mass of material was taken from the core area of the samples.

## 3. Results and Discussions

**Figure 3:**
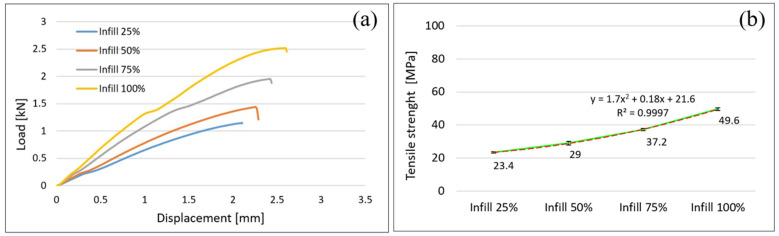
Tensile properties of 3D FFF samples. (**a**) load-displacement curves; (**b**) relationship between tensile strength and infill density.

**Figure 4:**
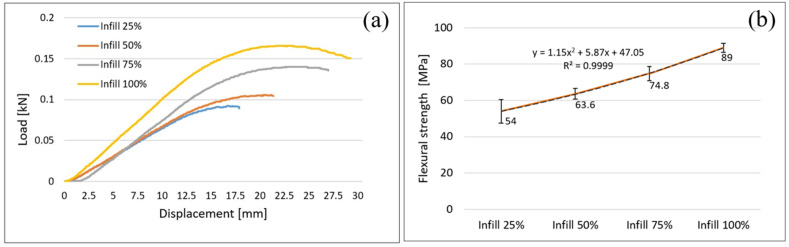
Three-points flexural tests; (**a**) flexural load—displacement curves; (**b**) infill density influence on flexural strength.

**Figure 5:**
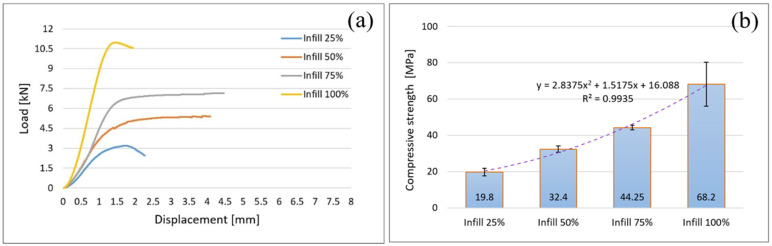
Compression test results for samples with rectangular cross-section; (**a**) load—displacement curves; (**b**) relationship between infill density and compression strength.

As it is being shown in previous studies the ability of AMed parts to bear loads depend on the manufacturing parameters and filament characteristics such as infill density (ID), the volume faction of fiber to matrix of the filament, interlayer cohesion, voids volume in the structures of AMed parts, bead-bead interface bonding etc. Changes in infill density lead to structural changes and obviously influence the strength of AMed fabricated parts. The data presented in this paper for PLA reinforced with glass fiber shows that the both tensile, flexural and compression strength increase with infill density as shown in Figure 3, Figure 4 and Figure 5.

Increasing the infill density can lead to decreased gaps within the structure (i.e., an increase in the material content), increase the number of the filament junctions and improve interlayer and bead-bead cohesion of the AMed material by FFF process. Therefore, this may lead to increase the strength of AMed part and they can bear a higher load.

### 3.1. Tensile Properties of 3D FFF Samples

Figure 3 illustrates load-displacement curves (Figure 3a) and tensile strength (Figure 3b), as an average of the values for the 5 samples tested, for the four different infill densities (25%, 50%, 75%, and 100%). It can be seen that infill density considerable influence the tensile properties. The tensile strength increases with infill density as shown in Figure 3b. As it is evident, the tensile strength (49.6 MPa, with a standard deviation of ±0.8 MPa) of the samples with a 100% infill density is approximately two times higher than of the samples with 25% infill density (23.4 MPa, with ±0.49 standard deviation). This observation is consistent with those published in [19,22,23,27,35,40,47].

Relationship between tensile strength and infill density is express by a polynomial equation of order 2 (Figure 3b, red line). The polynomial equation was chosen because the value of R2 is 0.9997 which more accurately describes this relationship than a linear one with R2 of 0.9699.

### 3.2. Flexural Properties of FFF Samples

Results of three-points flexural (bending) tests of the samples with 25%, 50%, 75%, and 100% infill percentage in Figure 4 are shown. Load-displacement curves (Figure 4a) follows the same trend as was for the tensile namely the strength increase as the infill density of samples increases. Thus, samples with 100% ID support the highest load (0.17 kN) while the samples with 25% failed under a maximum load of 0.09 kN. The average values of the flexural strength for the four infill densities are shown in Figure 4b. As it is evident, the samples with 100% infill density have the highest flexural strength (89 MPa, with ±2.45 MPa standard deviation). Increasing the ID from 25% to 100% led to increase the flexural strength of 35%, which means that infill density has a major influence on flexural properties as also found in previous studies [22,23,24,37,45]. In Figure 4b (dark blue line), the relationship between the flexural strength and the infill density is also indicated by an order 2 polynomial.

### 3.3. Compression Properties of FFF Samples and Fuselage Sections

A standard for AMed polymers (reinforced or not), by FFF process, has not been developed yet and therefore, in this paper, the ASTM D695 for classical polymers has been used [38,42]. This standard indicates that the samples should be in the form of a right cylinder with the cross-section diameter equal to 12.7 mm or a square prism with the cross-section of 12.7 mm by 12.7 mm. The length of samples depends on the mechanical properties under investigation and it is expressed in function on slenderness ratio. Samples’ length must be twice the cross-section, if compressive yield and strength are determined. If compressive elastic modulus is of interest the slenderness ratio must be between 11 and 16 [35,42]. The aim of the present paper is to investigate the behavior of material and influence of infill density on mechanical properties of the samples made of Filaticum PLA-GF and fabricated by FFF process. Therefore, they were designed, fabricated and compression tested samples of square prism shape. Samples were manufactured by FFF process with an infill density of 25%, 50%, 75%, and 100%. 5 samples of each type of infill density were AMed and subjected to compression tests. The data obtained during the compression tests were processed and the load-displacement curves, as an average of the values for the 5 samples tested of each infill type, are shown in Figure 5a.

The load-displacement curves (Figure 5a) clearly show that the infill density influences the behavior of the compression load. The samples with the lowest percentage of infill, 25%, bear the lowest stress (Figure 5b) and therefore have the lowest compressive strength, 19.8 MPa (with ±2.04 MPa standard deviation). Increasing the infill density has as a result the supporting of higher compression loads and implicitly higher strength since the inner void spaces were reduced. This statement is supported by the values plotted in Figure 5. As evident, the samples with infill density of 100% withstand the highest load, of 10.97 kN (Figure 5a), having a maximum compressive strength of 68.2 MPa (with standard deviation of ±12.02 MPa) (48.5% higher than the samples with infill of 25%) (Figure 5b). These findings are consistent with previous studies [21,26,34,35,36,37]. The samples AMed with 50% and 75% show lower compressive strength than with 100% (Figure 5), but among the four types of filling percentages, they show more ductile behavior. Also, the areas under the curves are greater than those for 25% and 100% which indicate that samples with 50% and 75% infill density can accumulate more energy before fractures and therefore they are more toughness. This observation is in line with [20].

As previously emphasized, the present work aims to investigate the mechanical performances of 3D FFF elements made of PLA reinforced with short glass fiber in order to evaluate the mechanical and thermal behavior and its potential use in structures that are part of a UAV. Although structural elements fabricated by FFF process are not common in the literature because of the difficulties in evaluating the mechanical properties [38], in the present work, the authors fabricated by FFF process and subjected to mechanical compression tests fuselage sections that are included in the structure of the UAV fuselage. The conception, design and manufacture of the UAV components using additive manufacturing technologies are entirely carried out by the authors of this work. It is worth emphasizing that the mentioned UAV is the major objective of a research project developed by the authors. Therefore, the concern of the authors to analyze both the materials used in the FFF process of the structural elements and the influence of the different AM parameters used in their manufacture by FFF is justified.

It is well known that the fuselage is one of the major aircraft components and connects the other parts of the aircraft and its skin (shell) withstands the loads generated by them (wings, landing gear, empennage, engines etc.) but also those from the internal pressurization of fuselage [48]. The shell structure (or skin) is then strengthened using a series of frames and stringers [48,49]. To form the complete fuselage, large tubular sections are made which are then joined with fasteners. Stringers are stiffening elements with the role of carrying the stresses. They should be able to carry both a tensile and compressive forces and increase the resistance of the fuselage skin to deformation [50]. The cylindrical or conical shells are usually manufactured from composites and carries tension, compression and shear loads [48,49,51,52].

**Figure 6:**
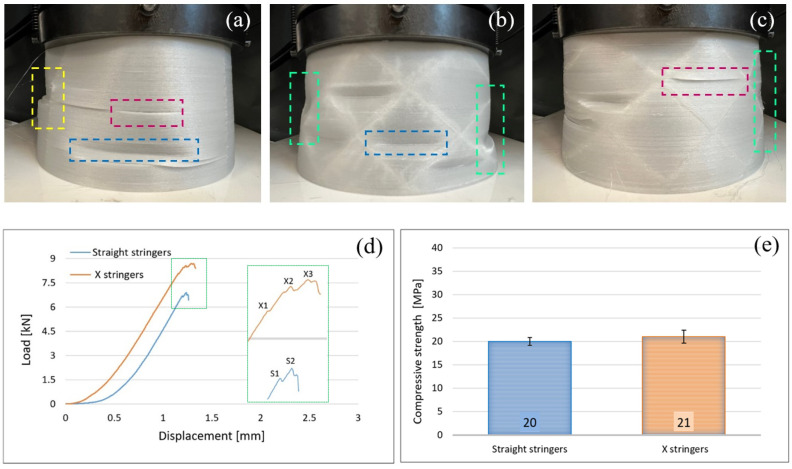
Compression tests of UAV fuselage sections. Fuselage section with straight (**a**) and X stringers (**b**,**c**), respectively during compression tests; (**d**) load-displacement curves; (**e**) compression strength of straight and X stringers.

As previous demonstrated (Figure 3, Figure 4 and Figure 5), the use of an infill density of 100% in the manufacture of PLA components reinforced with glass fiber by the FFF process can ensure the best mechanical performance. Therefore, the fuselage sections, and implicitly the entire fuselage of the UAV, were manufactured by FFF using an infill density of 100%. Regarding the stiffening of the fuselage skin, two types of stringers orientations were used: stringers oriented longitudinally (straight/longitudinal stringers, without inclination angle) (Figure 1a and Figure 6a) and stringers arranged at an angle of ±45° (Figure 1b and Figure 6b). In this paper, to analyze the behavior of the developed UAV fuselage under mechanical loads, the stiffened conical fuselage sections were subjected to an axial compressive load. Typically, the most important failure criteria for these structures are stress analysis and buckling [49,53]. Regarding the buckling failure modes of stiffened composite cylindrical/conical shells can be subdivided as global buckling, local skin buckling, and stiffener crippling [49,53,54]. Among these failure modes, the global buckling is the most frequent mode for composite shells subjected to axial compression [53].

The recorded load-displacement data for the stiffened conical shell are processed and average load–displacement plots are obtained (Figure 6d). Load-displacement curves (Figure 6d) illustrate that the PLA-GF fuselage sections stiffened with X stringers withstand a compression load higher than those with straight stringers. The sections with X stringers begin to fail under the action of a compression force of 8.45 kN (S1 peak), while those with straight stringers at a load of 6.68 kN (X1 peak). The graphs in Figure 6e show that the fuselage sections with X and straight stringers have very close values of compressive strength, 21 MPa (±1.41 MPa standard deviation) and 20 MPa (±0.82), respectively. In the Figure 6a, it is remarked a local distortion (blue dashed line area) in the lower part of the fuselage sections stiffened with straight/longitudinal stringers and then under axial compression the longitudinal stringers failed (the area marked with a yellow dashed line), delamination between the layers appears (the area marked with a red dashed line) and the structure globally fails. The fuselage sections stiffened with X stringers (Figure 6b,c), under axial compression load, has a distortion like sinusoidal shape (Figure 6b, green dashed line areas). It can be seen that just in certain areas in the shell the buckling is appeared (local skin buckling) (Figure 6b, blue dashed line areas), these areas present high distortions comparing with the other shell places which are not affected by buckling. This compression behavior of stiffened shell is line with that mentioned in the papers [49,53]. Three peaks (X1–X3) can be observed on the load-displacement curve of the fuselage sections stiffened with X stringers and two peaks (S1, S2), respectively, on the load-displacement curve of the fuselage sections stiffened with straight/longitudinal stringers (Figure 6d). It is considered that peaks X1 (8.45 kN) and S1 (6.68 kN) are caused by the initial damage in the shell (local buckling). After local damage, as the stress distributes, the fuselage section can sustain a higher load, which in turn can cause further damage in various ways until the applied load reaches the second peak at about 8.56 kN (X2) for sections with stringers at ±45°, and respectively 6.9 kN (S2) for those with straight stringers. As the load increases, in the case of sections with straight stringers, the skin breaks, the layers’ delamination appears and also the stringers break, which means final rupture load is achieved at 6.9 kN (S2 peak). At the sections with stringers at ±45°, peak S2 is believed to be generated by local detachment of material layers (delamination) (Figure 6c, red dashed line areas), in addition to local buckling. The final failure load is achieved at 8.70 kN (X3 peak), a local buckling of the skin occurred near the lower end of the fuselage section. It is more than obvious that the manufacture of the fuselage sections with stringers arranged at ±45°, in addition to the use of an infill density of 100% in the manufacture by FFF, leads to obtaining structures with a high rigidity compared to those provided with longitudinal stringers reason for which they are used in the fabrication of the UAV fuselage shown in Figure 2a.

### 3.4. Thermal Properties of Filament and FFF Samples

**Figure 7:**
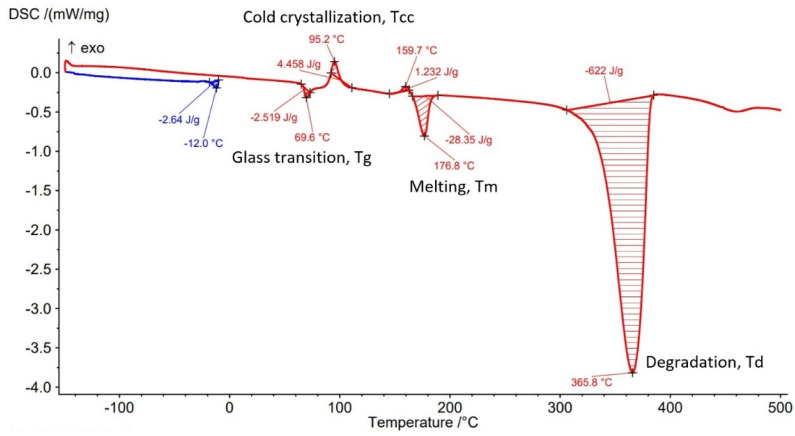
DSC curve for Filaticum PLA−GF filament.

**Figure 8:**
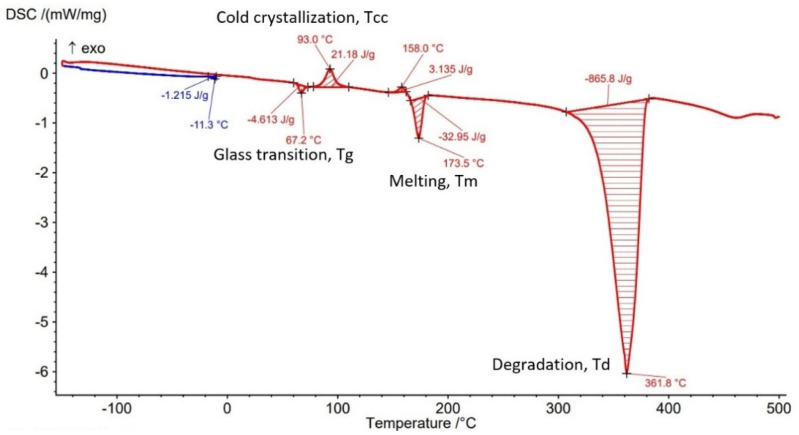
DSC results for sample with 25% infill density.

**Figure 9:**
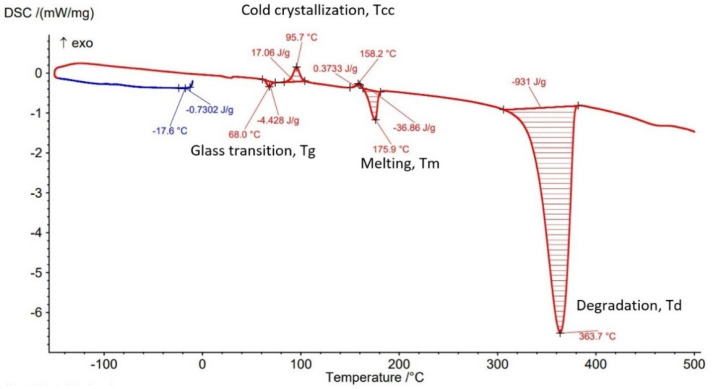
DSC thermogram for sample with 50% infill density.

**Figure 10:**
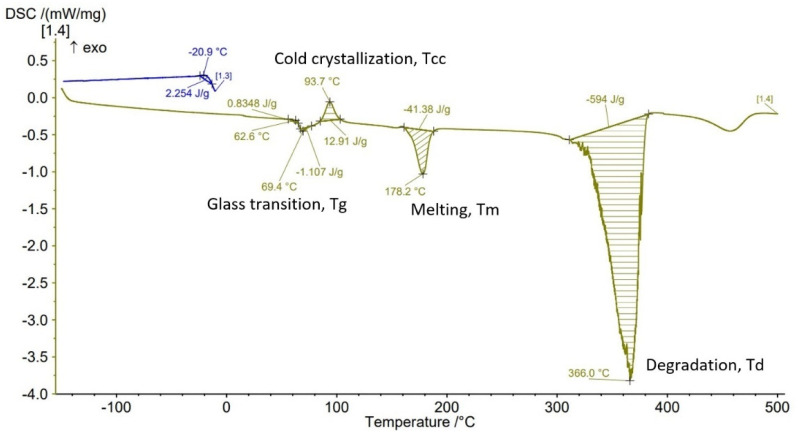
DSC curves related to sample with 75% infill density.

**Figure 11:**
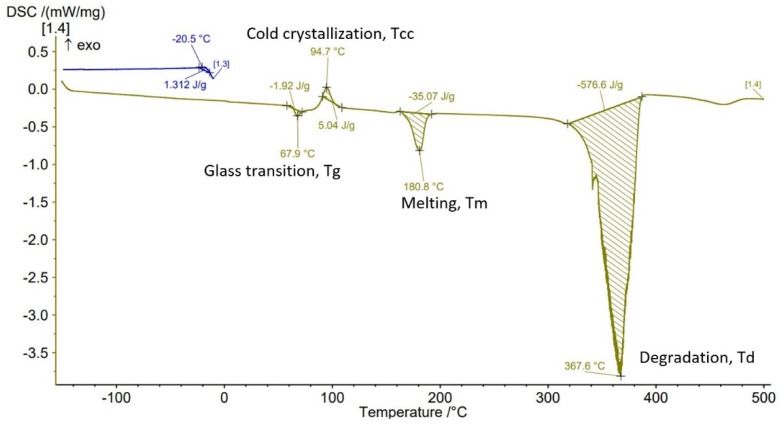
DSC thermogram related to sample with 100% infill density.

The Differential Scanning Calorimetry (DSC) curves of PLA-GF filament and AMed sample with different infill density in Figure 7, Figure 8, Figure 9, Figure 10 and Figure 11 are illustrated. It is worth emphasizing the fact that the Filaticum PLA-GF filament producer [43,44] only provides data on the glass transition temperature (Tg) of neat PLA (matrix of PLA-GF filament), but does not provide data on the thermal behavior of the material before and after extruding of PLA-GF filament. Glass transition temperature of neat PLA indicated by the manufacturer [43,44] is 50–60 °C. In previous studies [14,26,32,33] glass transition temperature (Tg) of neat PLA is 60–62 °C [26], about 58 °C [32,33] or 54 °C [14]. Therefore, in this paper, the glass transition temperature indicated by the manufacturer is considered as a reference for comparisons.

All DSC curves (Figure 7, Figure 8, Figure 9, Figure 10 and Figure 11) reveal four different transition peaks. The first transition (peak) represents the transition from the glassy phase to the rubbery one, that is glass transition temperature (Tg), the second peak that indicates an exothermic transition represents the cold crystallization temperature (Tcc), while the third transition, indicated by an endothermic peak, represents melting temperature (Tm). The fourth endothermic peak represents the total degradation temperature of the material (Td). The DSC parameters values of the PLA-GF filament and samples manufactured by FFF are included in Table 3. As can be seen in Figure 7, Figure 8, Figure 9, Figure 10 and Figure 11 and Table 3, there is a change in the Tg of PLA after the incorporation of glass fiber (69.1 °C) compared to the Tg of neat PLA indicated by the manufacturer (50–60 °C). It is well known [6,55] that near the Tg of polymer composite materials, the molecular chains have greater flexibility and consequently have greater mobility. The increase in Tg of PLA after reinforcing with the glass fiber (Table 3, PLA-GF filament, 69.1 °C) suggests that the incorporation of the fiber restricts the mobility of the molecular chains in the PLA-GF composite. It is possible that this is due to a good bonding between PLA and glass fiber.

It is known that PLA, as a semicrystalline polymer material, has a low crystallization capacity. Therefore, the addition of fillers into semicrystalline matrices could determine a right or left shift of the crystallization temperature (Tcc), depending on the nucleation capacity of the filler. The appearance of exothermic peaks (Figure 7, Figure 8, Figure 9, Figure 10 and Figure 11), immediately after the glass transition temperature, which represents the crystallization temperature (Tcc), indicates that the incorporation of GF can influence the nucleation process and allows crystallization to take place immediately after cooling. The Tcc value slightly decreased compared to the PLA-GF filament as the filling density increased, which might be due to the effect of glass fiber nucleation on the crystallization of the material.

According to previous studies [32,33,56], the melting temperatures (Tm) for PLA are found in the 130–160 °C range (although higher values, up to 180 °C, have been reported, in the literature). However, as can be seen in Figure 7, Figure 8, Figure 9, Figure 10 and Figure 11 and Table 3, the presence of glass fiber in the PLA matrix generates the Tm peak shift to the right side of the curve reaching 176.8 °C, for PLA-GF filament, and between 173.5–180.8 °C for samples AMed with infill density from 25–100%.

In Figure 7, Figure 8, Figure 9, Figure 10 and Figure 11, it can be observed that the DSC thermograms show a small crystallization peak prior to the melting peak which is attributed to the transformation of unperfected crystals into more perfect crystals. From the DSC curves (Figure 7, Figure 8, Figure 9, Figure 10 and Figure 11) it can be seen that exceeding the melting temperatures (Tm) triggers the degradation process of the material, the maximum temperatures of complete degradation (Td) being highlighted by endothermic peaks. Comparing the data presented in Table 3 and DSC thermograms of the samples with infill density of 25%, 50%, 75%, and 100% respectively, it can be seen that the glass transition temperature (Tg) increases as the infill density increases, except the samples with infill density of 100% for which a lower Tg temperature was recorded than for samples with 75% infill density. The lowest Tg temperature was recorded for the samples with infill density of 25% (63.8 °C).

As previously mentioned, due to the incorporation of glass fibers in the PLA matrix, the glass transition temperature (Tg) shifted to a higher temperature. The increase in infill density determines an increase in the volume of deposited material and implicitly in the volume of glass fibers which can lead to a decrease in the mobility of the molecular chains of the matrix. The recording of samples with 75% infill density of a higher Tg than those with 100%, might be derived from higher glass fiber content of the material taken from samples with 75% for DSC analysis. This behavior is consistent with the studies published in [14]. It can also be noted that all glass transition temperatures of samples with the four types of infill densities are lower than the Tg of the filament (69.1 °C). Glass transition temperatures were approximately the same to that reported in [32,57]. Changing the infill density also influences the melting temperature, Tm, thus as infill density increases from 25% to 100%, the melting temperature increases from 173.5 °C to 180.8 °C. Therefore, for the samples with 100% ID, the highest melting temperature of 180.8 °C was recorded, as well as the highest temperature to reach complete degradation, 367.6 °C. It can be concluded that infill density, as a FFF parameter, influences mechanical properties but the thermal behavior of PLA-GF composite as well.

**Figure 12:**
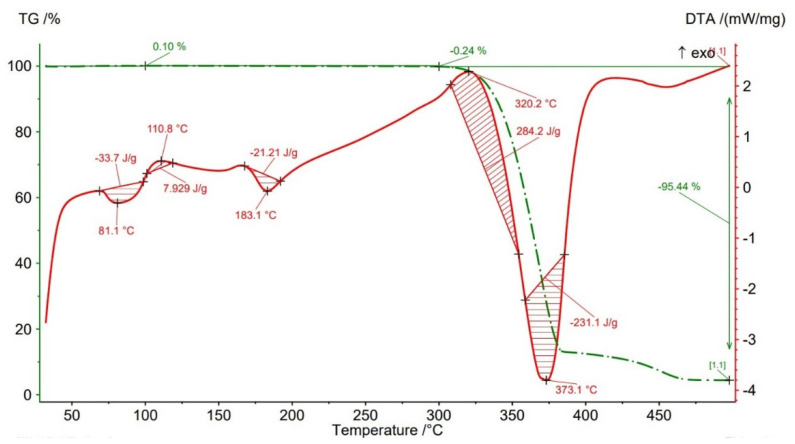
Thermogravimetric analysis results for Filaticum PLA−GF filament.

**Figure 13:**
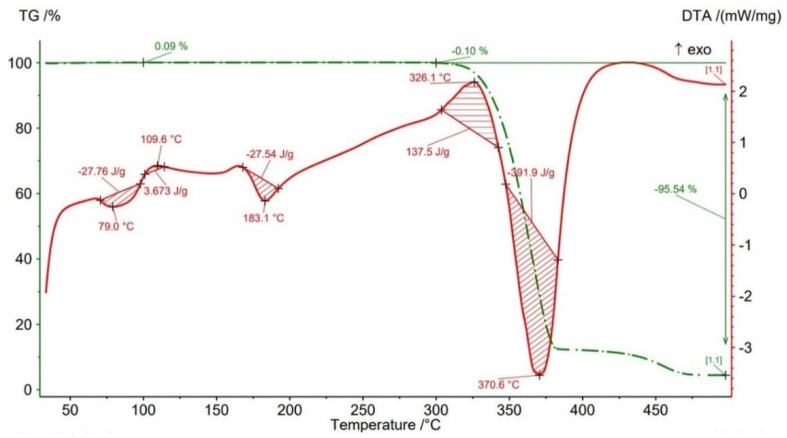
TGA results for sample with 25% infill density.

**Figure 14:**
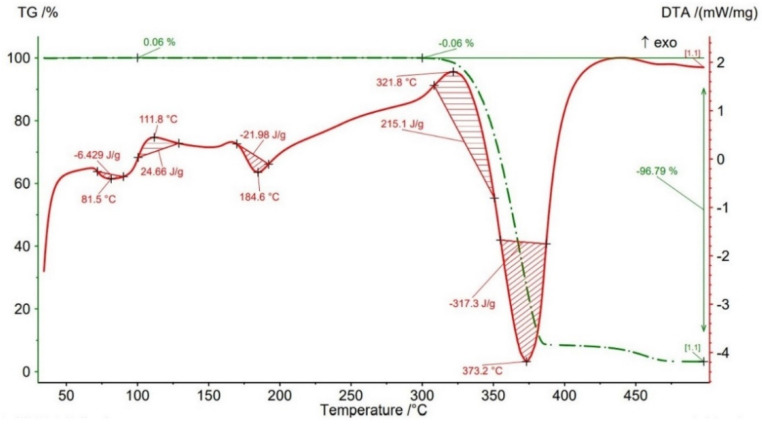
TGA results for sample with 50% infill density.

**Figure 15:**
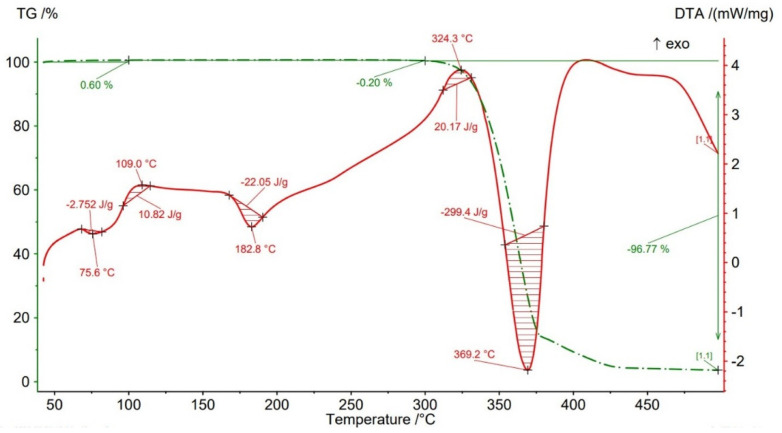
Thermogravimetric curves for sample with 75% infill density.

**Figure 16:**
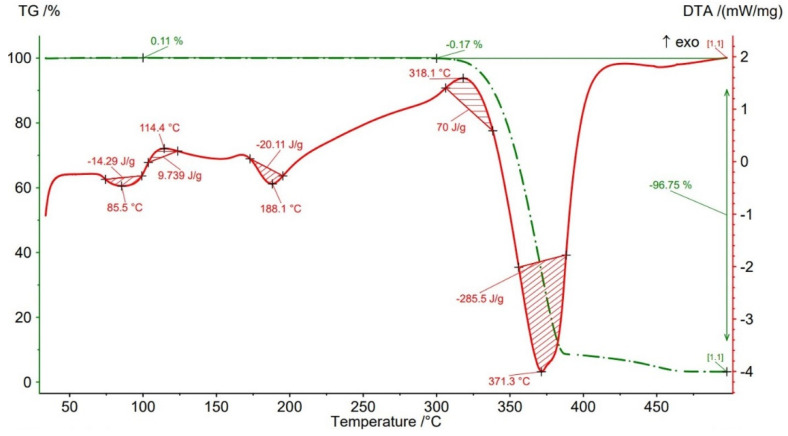
Thermogravimetric analysis curves for sample with 100% infill density.

Thermogravimetric analysis (TGA) results are presented in Figure 12, Figure 13, Figure 14, Figure 15 and Figure 16 and Table 4 both for Filaticum PLA-GF filament and FFF sample.

It can be seen that up to a temperature of 100 °C all the AMed samples as well as the filament show increases in weight. This increase in mass can be caused by the absorption of moisture by the PLA matrix, it being known that PLA has a high affinity to the humidity in the environment (PLA is hygroscopic material) [56]. The highest mass increase was recorded for the samples manufactured with an infill density of 75% (+0.6%) and 100% (+0.11%). One explanation for this behavior is that a higher infill density implies a higher volume of polymer matrix (PLA) and, consequently, a higher volume of absorbed moisture. However, in the samples with 75% infill density the weight gain is much higher than in those with 100% infill, a fact that can be attributed to the uneven distribution of the glass fiber in the base matrix and/or the presence of voids, defects in the polymer mass. Consequently, it is possible that the area from where the material was taken for TG analysis contained a lower volume of glass fiber but a higher volume of polymeric matrix which, as previously mentioned, absorbed a larger amount of moisture. Or, in the case of the samples with 100% infill density, the material sampling area contained a higher volume of fibers and/or voids and therefore a lower volume of PLA matrix. In the temperature range 100–300 °C (Figure 12, Figure 13, Figure 14, Figure 15 and Figure 16), both the material of the filament and the samples have a mass loss caused by the evaporation of moisture, the start of the thermal degradation process (burning) as the temperature exceeds 200 °C. These results are consistent with [57,58]. Filaticum PLA-GF filament (Figure 12) and samples manufactured with 75% infill density (Figure 15) recorded the highest mass loss, of 0.24% and 0.20%, respectively. From the analysis of the thermograms in Figure 12, Figure 13, Figure 14, Figure 15 and Figure 16, it is found that exceeding the temperature of 300 °C determines the increase in the thermal degradation of the polymer matrix both for the filament and for the FFF samples and also significant mass losses [56].

The thermogram of Filaticum PLA-GF filament (Figure 12) illustrates that the loss of thermal stability begins at a temperature of about 311 °C and above this temperature the accelerated degradation process begins with the burning of the material, indicated by the exothermic peak at 320.2 °C, until its complete degradation occurs at the temperature of 373.1 °C (endothermic peak) where the mass loss is 95.44%. Generally, the increase in the temperature of decomposition (degradation) determines an increase in the thermal stability of the material. Increasing the content of GF determines that the thermal degradation temperature of PLA-GF composites to tend slowly towards a high temperature [14,58].

The thermograms related to the samples manufactured by FFF (Figure 13, Figure 14, Figure 15 and Figure 16, Table 4) illustrate the fact that as the infill density increases, there is a slight increase in the thermal stability and the total degradation temperature. For the AMed material, the loss of thermal stability begins at a temperature of about 309.5 °C, for sample 75% infill density, 311.5 °C, for 25%, and 312.5 °C for 50% and 100% infill density, respectively. Regarding the infill density of 75%, the beginning of the thermal stability loss (about 309.5 °C) and the complete degradation (369.2 °C) at the lowest temperature among the four types of densities can be attributed to the presence in the mass of material subjected to the analysis of a lower volume of glass fibers, the presence of a higher number of manufacturing defects (voids) or a weak bond between the matrix (PLA) and the glass fiber. These observations are in line with the works [14,58].

## 4. Conclusions

This article reported the research on mechanical (tensile, flexural, and compression) and thermal properties of Filaticum PLA-GF composites used in the manufacture of components of an unmanned aerial vehicle (UAV) using the FFF process. In the current paper, the authors focused on the analysis of the influence of infill density, as an important process parameter in the FFF additive manufacturing technology, on the mechanical performance and thermal behavior of the samples and functional structures of fuselage sections type.

From the current study, based on the obtained results, the following conclusions can be drawn:

- It is highlighted that the infill density influences the mechanical properties of the components made of Filaticum PLA-GF composite, manufactured by FFF process, and to some extent also the thermal behavior.
- As the infill density increases, strength to tensile, flexural and compressive loading increases. Therefore, the sample with 100% infill density provides the highest mechanical characteristics even though the AM time and material consumption are higher than in the case of using a lower infill density.
- It is observed that the samples fabricated of Filaticum PLA-GF composite with 50% and 75% infill density present a higher toughness but, in the same time, a lower compressive strength than samples with 100% infill percentage.
- Since the manufacture of components with infill density of 100%, made of Filaticum PLA-GF composite, ensures high mechanical performance (confirmed by the results obtained after the mechanical tests), it was used in the manufacture of the structural elements that are part of a UAV (Figure 2). Therefore, conical sections from the UAV fuselage were manufactured and subjected to compression tests. The obtained results showed that the fuselage sections made of Filaticum PLA-GF composite and reinforced with stringers oriented at an angle of ±45° present better mechanical performances than those with longitudinal stringers.
- Because of the manufacturer does not provide data on the thermal behavior of Filaticum PLA-GF filament, glass transition (Tg), crystallization (Tcc), and melting (Tm) temperatures they were determined by DSC analyses (Figure 7, Figure 8, Figure 9, Figure 10 and Figure 11, Table 3). The results highlight that the addition of glass fiber in the PLA matrix causes the glass transition (Tg) to move to a higher temperature, of 69.1 °C, compared to the glass transition of neat PLA, of 50–60 °C.
- It was found that the glass transition (Tg), and melting (Tm) temperature increases slightly as the infill density increases.
- Thermogravimetric analysis (TGA) reveals the fact that for Filaticum PLA-GF filament the loss of thermal stability begins at a temperature of about 311 °C and its complete destruction occurs at a temperature of 373.1 °C with a mass loss of 95.44%.
- It was observed that the increase in infill density leads to a slight increase in thermal stability and complete degradation temperature. For samples with 25% infill density, the loss of thermal stability begins at a temperature of about 311.5 °C and for those with 100% infill density at 312.5 °C.

It is obvious that the FFF process parameters, particularly infill density, can significantly influence the mechanical properties but also the thermal behavior of AMed components. The results presented in this paper will help readers to understand the mechanical, thermal and degradation performance of commercial Filaticum PLA-GF filament. The present paper can be considered a starting point for investigating the properties of Filaticum PLA-GF filament, therefore further analyses are required to fully characterize its mechanical properties.

## Figures and Tables

**Table 1:** Dimensions of fuselage section and stringers subjected to compression test.

Dimension	Fuselage Section	Stringers
Base diameter [mm]	160	
Top diameter [mm]	140	
Fuselage section height [mm]	80	
Shell thickness [mm]	1	
Stringers thickness [mm]		1
Stringers height [mm]		4

**Table 2:** Manufacturing parameters for the PLA-GF composite samples and fuselage sections.

Parameter	Value		Unit
	Samples	Fuselage Sections	
Filament diameter	2.85		[mm]
Layer height	0.2	0.2	[mm]
Infill density	25; 50; 75; 100	100	[%]
Print speed	50	50	[mm/sec]
Extrusion temperature	250	250	[°C]
Building plate temperature	70	70	[°C]
Nozzle diameter	0.6	0.6	[mm]
Number of lower and upper layers	5	5	
Number of shell contours	2	2	
Infill pattern	Rectangular (0°/45° with respect to shell contour)		degree

**Table 3:** Differential Scanning Calorimetry (DSC) results for PLA-GF filament and AMed samples.

Samples	Glass Transition Temperature Tg [°C]	Cold Crystallization Temperature Tcc [°C]	Melting Temperature Tm [°C]	Full Degradation Temperature Td [°C]
Filament Filaticum PLA-GF	69.1	95.2	176.8	365.8
Sample 25% ID	63.8	93.0	173.5	361.8
Sample 50% ID	64	95.7	175.9	363.7
Sample 75% ID	65.7	93.7	178.2	366.0
Sample 100% ID	64.2	94.7	180.8	367.6

**Table 4:** Thermal stability (Tonset) and degradation peak (Td) temperatures and mass loss of Filaticum PLA-GF filament and FFF samples.

Samples	Thermal Stability Tonset	Peak of Burning Tb	Peak of Degradation Td	Mass Loss 20–100 °C	Mass Loss 100–300 °C	Mass Loss 300–500 °C
	[°C]	[°C]	[°C]	[%]	[%]	[%]
Filament Filaticum PLA-GF	311	320.2	373.1	+0.10	−0.24	−95.44
Sample 25% ID	311.5	326.1	370.6	+0.09	−0.10	−95.54
Sample 50% ID	312.5	321.8	373.2	+0.06	−0.06	−96.79
Sample 75% ID	309.5	324.3	369.2	+0.60	−0.20	−96.77
Sample 100% ID	312.5	318.1	371.3	+0.11	−0.17	−96.75

## Data Availability

Not applicable.

## References

[1] (2021). Additive Manufacturing—General Principles—Fundamentals and Vocabulary..

[2] Billah K.M., Lorenzana F., Martinez N.L., Chacon S., Wicker R.B., Espalin D. Thermal analysis of thermoplastic materials filled with chopped fiber for large area 3d printing. Proceedings of the Solid Freeform Fabrication 2019: Proceedings of the 30th Annual International Solid Freeform Fabrication Symposium—An Additive Manufacturing Conference.

[3] Forés Garriga A., Pérez M., Gómez-Gras G., Reyes G. (2020). Role of infill parameters on the mechanical performance and weight reduction of PEI Ultem processed by FFF. Mater. Des..

[4] Chao H., Qing-Hua Q. (2020). Advances in fused deposition modeling of discontinuous fiber/polymer composites. Curr. Opin. Solid State Mater. Sci..

[5] Wang G., Zhang D., Li B., Wan G., Zhao G., Zhang A. (2019). Strong and thermal-resistance glass fiber-reinforced polylactic acid (PLA) composites enabled by heat treatment. Int. J. Biol. Macromol..

[6] Wang G., Zhang D., Wan G., Li B., Zhao G. (2019). Glass fiber reinforced PLA composite with enhanced mechanical properties, thermal behavior, and foaming ability. Polymer.

[7] Ruz-Cruz M.A., Herrera-Franco P.J., Flores-Johnson E.A., Moreno-Chulim M.V., Galera-Manzano L.M., Valadez-González A. (2022). Thermal and mechanical properties of PLA-based multiscale cellulosic biocomposites. J. Mater. Res. Technol..

[8] Facca A.G., Kortschot M.T., Yan N. (2007). Predicting the tensile strength of natural fibre reinforced thermoplastics. Compos. Sci. Technol..

[9] Xiu H., Qi X., Liu Z., Zhou Y., Bai H., Zhang Q., Fu Q. (2016). Simultaneously reinforcing and toughening of polylactide/carbon fiber composites via adding small amount of soft poly(ether)urethane. Compos. Sci. Technol..

[10] Jing M., Che J., Xu S., Liu Z., Fu Q. (2018). The effect of surface modification of glass fiber on the performance of poly(lactic acid) composites: Graphene oxide vs. silane coupling agents. Appl. Surf. Sci..

[11] Yu B., Geng C., Zhou M., Bai H., Fu Q., He B. (2016). Impact toughness of polypropylene/glass fiber composites: Interplay between intrinsic toughening and extrinsic toughening. Compos. Part B Eng..

[12] Karger-Kocsis J., Mahmood H., Pegoretti A. (2015). Recent advances in fiber/matrix interphase engineering for polymer composites. Prog. Polym. Sci..

[13] Jaszkiewicz A., Bledzki A.K., Franciszczak P. (2013). Improving the mechanical performance of PLA composites with natural, man-made cellulose and glass fibers—A comparison to PP counterparts. Polimery.

[14] Huda M.S., Drzal L.T., Mohanty A.K., Misra M. (2006). Chopped glass and recycled newspaper as reinforcement fibers in injection molded poly(lactic acid) (PLA) composites: A comparative study. Compos. Sci. Technol..

[15] Deniz Varsavas S., Kaynak C. (2018). Effects of glass fiber reinforcement and thermoplastic elastomer blending on the mechanical performance of polylactide. Compos. Commun..

[16] Chacón J.M., Caminero M.A., García-Plaza E., Núñez P.J. (2017). Additive manufacturing of PLA structures using fused deposition modelling: Effect of process parameters on mechanical properties and their optimal selection. Mater. Des..

[17] Gómez-Gras G., Jerez-Mesa R., Travieso-Rodriguez J.A., Lluma-Fuentes J. (2018). Fatigue performance of fused filament fabrication PLA specimens. Mater. Des..

[18] Aloyaydi B., Sivasankaran S., Hany A. (2019). Influence of infill density on microstructure and flexural behavior of 3D printed PLA thermoplastic parts processed by fusion deposition modeling. AIMS J..

[19] Pandžić A., Hodzic D., Milovanović A., Katalinic B. (2019). Effect of infill type and density on tensile properties of PLA material for FDM process. Proceedings of the 30th DAAAM International Symposium.

[20] Ma Q., Rejab M., Kumar A.P., Fu H., Kumar N.M., Tang J. (2021). Effect of infill pattern, density and material type of 3D printed cubic structure under quasi-static loading. Proc. Inst. Mech. Eng. Part C J. Mech. Eng. Sci..

[21] Hodzic D., Pandzic A. (2021). Influence of Infill Design on Compressive and Flexural Mechanical Properties of FDM Printed PLA Material. Proceedings of the 32nd DAAAM International Symposium.

[22] Gonabadi H., Yadav A., Bull S.J. (2020). The effect of processing parameters on the mechanical characteristics of PLA produced by a 3D FFF printer. Int. J. Adv. Manuf. Technol..

[23] Dave H.K., Patadiya N.H., Prajapati A.R., Rajpurohit S.R. (2019). Effect of infill pattern and infill density at varying part orientation on tensile properties of fused deposition modeling-printed poly-lactic acid part. Proc. Inst. Mech. Eng. Part C J. Mech. Eng. Sci..

[24] Akhoundi B., Behravesh A.H. (2019). Effect of filling pattern on the tensile and flexural mechanical properties of FDM 3D printed products. Exp. Mech..

[25] Wang S., Ma Y., Deng Z., Zhang S., Cai J. (2020). Effects of fused deposition modeling process parameters on tensile, dynamic mechanical properties of 3D printed polylactic acid materials. Polym. Test.

[26] Abeykoon C., Sri-Amphorn P., Fernando A. (2020). Optimization of fused deposition modeling parameters for improved PLA and ABS 3D printed structures. Int. J. Lightweight Mater. Manuf..

[27] Cwikła G., Grabowik C., Kalinowski K., Paprocka I., Ociepka P. (2017). The influence of printing parameters on selected mechanical properties of FDM/FFF 3D-printed parts. IOP Conference Series: Materials Science and Engineering. Proceedings of the ModTech International Conference—Modern Technologies in Industrial Engineering.

[28] Singh Mehta L., Pillai P. (2017). Compression Testing of PLA in 3D Printing. Int. J. Electron. Electr. Comput. Syst..

[29] Mercado-Colmenero J.M., Rubio-Paramio M.A., la Rubia-Garcia M.D., Lozano-Arjona D., Martin-Doñate C. (2019). A numerical and experimental study of the compression uniaxial properties of PLA manufactured with FDM technology based on product specifications. Int. J. Adv. Manuf. Technol..

[30] Abbas T., Othman F.M., Ali H.B. (2017). Effect of infill Parameter on compression property in FDM Process. Int. J. Eng. Res. Appl..

[31] Pepelnjak T., Karimi A., Maček A., Mole N. (2020). Altering the Elastic Properties of 3D Printed Poly-Lactic Acid (PLA) Parts by Compressive Cyclic Loading. Materials.

[32] Lim L.-T., Auras R., Rubino M. (2008). Processing technologies for poly(lactic acid). Prog. Polym. Sci..

[33] Carrasco F., Pagès P., Gámez-Pérez J., Santana O.O., Maspoch M.L. (2010). Processing of poly(lactic acid): Characterization of chemical structure, thermal stability and mechanical properties. Polym. Degrad. Stab..

[34] Subramaniyan M., Karuppan S., Prabanjan P., Pugazh A., Pynthamizh A. (2022). Survey on compression property of sandwich 3D printed PLA components. Mater. Today Proc..

[35] Torre R., Brischetto S., Dipietro I.R. (2021). Buckling developed in 3D printed PLA cuboidal samples under compression: Analytical, numerical and experimental investigations. Addit. Manuf..

[36] Zhu Y., Gao Y., Jiang J., Gu H., Lv S., Ni H., Wang X., Jia C. (2019). Study on effects of FFF 3D printing parameters on mechanical properties of polylactic acid. IOP Conf. Ser. Mater. Sci. Eng..

[37] Baich L., Manogharan G., Marie H. (2015). Study of infill print design on production cost-time of 3D printed ABS parts. Int. J. Rapid Manuf..

[38] Brischetto S., Torre R. (2020). Tensile and Compressive Behavior in the Experimental Tests for PLA Specimens Produced via Fused Deposition Modelling Technique. J. Compos. Sci..

[39] (2014). Standard Test Method for Tensile Properties of Plastics.

[40] Chicos L.-A., Pop M.A., Zaharia S.-M., Lancea C., Buican G.R., Pascariu I.S., Stamate V.-M. (2022). Infill Density Influence on Mechanical and Thermal Properties of Short Carbon Fiber-Reinforced Polyamide Composites Manufactured by FFF Process. Materials.

[41] (2003). Standard Test Methods for Flexural Properties of Unreinforced and Reinforced Plastics and Electrical Insulating Materials1.

[42] (2015). Standard Test Method for Compressive Properties of Rigid Plastics.

[43] Filaticum Glass Reinforced. https://filaticum.com/en/product/philament-glass-reinforced/.

[44] Filaticum Comparision Datasheet. https://filaticum.com/wp-content/uploads/2021/03/filaticum.com-comparison-datasheet-2021-0308.pdf.

[45] (2021). Standard Test Method for Transition Temperatures and Enthalpies of Fusion and Crystallization of Polymers by Differential Scanning Calorimetry.

[46] (2020). Standard Test Method for Compositional Analysis by Thermogravimetry.

[47] Wang K., Xie X., Wang J., Zhao A., Peng Y., Rao Y. (2020). Effects of infill characteristics and strain rate on the deformation and failure properties of additively manufactured polyamide-based composite structures. Results Phys..

[48] Querin O.M., Toporov V.V., Liu D., Busch L.H., Hühne C., Niemann S., Kolesnikov B. (2014). Topology and Parametric Optimization of a Lattice Composite Fuselage Structure. https://altairuniversity.com/wp-content/uploads/2014/04/Topology_and_Parametric_Optimization-of-a-latice_Composite_Fuselage_Structure_as_published.pdf.

[49] Kanou H., Nabavi S., Jam J. (2013). Numerical modeling of stresses and buckling loads of isogrid lattice composite structure cylinders. Int. J. Eng. Sci. Technol..

[50] van Gent I., Kassapoglou C. (2014). Cost-weight trades for modular composite structures. Struct. Multidisc. Optim..

[51] Im J.-M., Shin K.-B. (2022). Study on the prediction method of onset and propagation of damage of unit composite lattice structure. J. Mech. Sci. Technol..

[52] Brischetto S., Ferro C.G., Maggiore P., Torre R. (2017). Compression Tests of ABS Specimens for UAV Components Produced via the FDM Technique. Technologies.

[53] Rafiee M., Hejazi M., Amoushahi H. (2021). Buckling response of composite cylindrical shells with various stiffener layouts under uniaxial compressive loading. Structures.

[54] Zarei M., Rahimi G.H., Hemmatnezhad M. (2021). On the buckling resistance of grid-stiffened composite conical shells under compression. Eng. Struct..

[55] Akindoyo J.O., Beg M.D.H., Ghazali S., Heim H.P., Feldmann M., Mariatti M. (2021). Simultaneous impact modified and chain extended glass fiber reinforced poly(lactic acid) composites: Mechanical, thermal, crystallization, and dynamic mechanical performance. J. Appl. Polym. Sci..

[56] Farah S., Anderson D., Langer R. (2016). Physical and mechanical properties of PLA, and their functions in widespread applications—A comprehensive review. Adv. Drug Deliv. Rev..

[57] Pillin I., Montrelay N., Bourmaud A., Grohens Y. (2008). Effect of thermo-mechanical cycles on the physico-chemical properties of poly(lactic acid). Polym. Degrad. Stab..

[58] Sun Y., Zheng Z., Wang Y., Yang B., Wang J., Mu W. (2022). PLA composites reinforced with rice residues or glass fiber—A review of mechanical properties, thermal properties, and biodegradation properties. J. Polym. Res..

